# *MYB-NFIB* gene fusions identified in archival adenoid cystic carcinoma tissue employing NanoString analysis: an exploratory study

**DOI:** 10.1186/s13000-019-0855-8

**Published:** 2019-07-13

**Authors:** John B. McIntyre, Jenny J. Ko, Jodi Siever, Angela M. Y. Chan, Roderick H. W. Simpson, Desiree Hao, Harold Y. Lau

**Affiliations:** 1 Translational Laboratory, Department of Oncology, University of Calgary, Calgary, AB Canada; 2 Department of Medical Oncology, BC Cancer – Abbotsford, Abbotsford, BC Canada; 3 Faculty of Medicine, Southern Medical Program University of British Columbia, Kelowna, BC Canada; 4 Translational Laboratory, Tom Baker Cancer Centre, Calgary, AB Canada; 5 Department of Anatomical Pathology, University of Calgary, Foothills Medical Centre, Calgary, AB Canada; 6 Department of Medical Oncology, Tom Baker Cancer Centre, University of Calgary, Calgary, AB Canada; 7 Department of Radiation Oncology, University of Calgary, Tom Baker Cancer Centre, Calgary, AB Canada

**Keywords:** Adenoid cystic carcinoma, Salivary gland, *MYB*, *MYB-NFIB*, NanoString, Fusion transcript

## Abstract

**Background:**

Adenoid cystic carcinoma (ACC) is a slow growing salivary gland malignancy that is molecularly characterized by t(6:9)(q22–23;p23–24) translocations which predominantly result in *MYB-NFIB* gene fusions in nearly half of tumours. Detection of *MYB-NFIB* transcripts is typically performed with fresh ACC tissue using conventional RT-PCR fragment analysis or FISH techniques, which are prone to failure when only archival formalin fixed paraffin embedded (FFPE) tissue is available. The purpose of this pilot study was to evaluate the utility of NanoString probe technology for the detection of *MYB-NFIB* transcripts in archival ACC tissue.

**Methods:**

A NanoString probeset panel was designed targeting the junctions of three currently annotated *MYB-NFIB* fusion genes as well as 5′/3′ *MYB* probesets designed to detect *MYB* gene expression imbalance. RNA isolated from twenty-five archival ACC specimens was profiled and analyzed. RT-qPCR and sequencing were performed to confirm NanoString results. MYB protein expression was analyzed by immunohistochemistry.

**Results:**

Of the 25 samples analyzed, 11/25 (44%) expressed a high degree of MYB 5′/3′ imbalance and five of these samples were positive for at least one specific *MYB-NFIB* variant in our panel. *MYB-NFIB* variant detection on NanoString analysis was confirmed by direct cDNA sequencing. No clinical correlations were found to be associated with *MYB* fusion status.

**Conclusion:**

We conclude that the application of NanoString digital probe counting technology is well suited for the detection and quantification of *MYB-NFIB* fusion transcripts in archival ACC specimens.

**Electronic supplementary material:**

The online version of this article (10.1186/s13000-019-0855-8) contains supplementary material, which is available to authorized users.

## Background

Adenoid cystic carcinoma (ACC) is a rare malignancy that can arise from head and neck sites such as salivary glands, nasopharynx, oropharynx, external ear, lacrimal gland; or other areas such as trachea, breast and female genitalia [1]. The disease is characterized by slow yet progressive tumour growth with a tendency for perineural invasion and local recurrence, often resulting in craniofacial infiltration. Primary treatment is surgical resection and/or radiotherapy however no effective systemic chemotherapeutic options are available at present [2–4]. Early cytogenetic studies of ACC demonstrated that recurrent loss of the terminal long arm of chromosome 6 as well as reciprocal translocations of chromosomes 6q and 9p are molecular hallmarks of the disease [5]. The t(6:9)(q22–23;p23–24) translocation was subsequently shown to result in the fusion of the *MYB* proto-oncogene with the nuclear transcription factor gene *NFIB* [6]. In a seminal paper, Persson et al were the first to report the presence of chimeric *MYB-NFIB* fusion transcripts in both breast and head and neck ACCs but total absence in non-ACC tumours [6]. Furthermore, they and others were able to identify 14 *MYB-NFIB* transcript variants by conventional RT-PCR methods [6] [7]. Approximately 50% of all ACCs studied to date have been shown to express *MYB-NFIB* chimeric transcripts suggesting a prominent role in ACC tumourgenecity [8].

Fluorescence *insitu* hybridization (FISH) and reverse transcriptase-PCR (RT-PCR) techniques have been the primary analytical methods employed to detect *MYB-NFIB* rearrangements in ACC to date. Generally, FISH assays are limited in that they are DNA-based and can only predict the potential for fusion transcript expression and offer minimal information regarding specific fusion breakpoints. Alternatively, RNA-based assays such as RT-PCR, are able to better characterize ACC by not only confirming the expression of *MYB-NFIB* transcripts but by also providing information regarding the fusion variants present in a tumour. However, in studies where RT-PCR is used to detect *MYB-NFIB* transcripts, the best results have been achieved when frozen tumour material is available for interrogation as the technique does not lend itself well to studies where formalin fixed paraffin embedded (FFPE) tissues may be the only samples available to researchers [9]. These shortcomings can limit the detection and annotation of specific *MYB-NFIB* fusion transcripts and their potential biological function in the context of tumour phenotype and behaviour. This importance is exemplified in another recent study by Mitani et al who observed distinct gene expression profiles in frozen ACC tumours based on the location of fusion breakpoints within *MYB* and the related *MYBL1* gene [10].

In our current study we sought to determine if *MYB* and *MYB-NFIB* transcript expression in archival FFPE head and neck adenoid cystic carcinomas could be detected using NanoString probe based methodology. NanoString technology employs unique digital colour-coded probes that hybridize directly to specific mRNA targets permitting the detection and quantified expression of multiple targets in a single reaction [11]. This robust technology does not require mRNA reverse transcription and thus holds a particular advantage over PCR-based methods that can suffer from polymerase inhibition and amplification bias [12]. Furthermore, NanoString methodology can overcome limitations imposed by fragmented and degraded RNA typical of FFPE tissues, in detecting fusion transcripts which hampers conventional amplicon-sizing RT-PCR assays like those used in previous studies where frozen tissues were required [6, 9, 13, 14].

## Methods

### Tissue samples and clinical data

After approval by the provincial research ethics board, 60 consecutive patients who were diagnosed with ACC and evaluated at the Tom Baker Cancer Centre (Calgary, Alberta) between January 1, 2007 and December 31, 2013, were identified. After chart review of the 60 subjects, we identified 25 patients with adequate tissue biopsies that were available for further study. Twenty-five formalin fixed paraffin embedded (FFPE) primary tumours were retrieved from the tissue archive. The patient, tumor characteristics and treatments are shown in Table 1. Normal paired salivary gland tissue was available from twelve patients. All patient samples underwent additional expert pathology review by RHWS to confirm the diagnosis of ACC. Patient and disease characteristics, as well as survival outcomes, were collected by chart review.

**Table 1:**
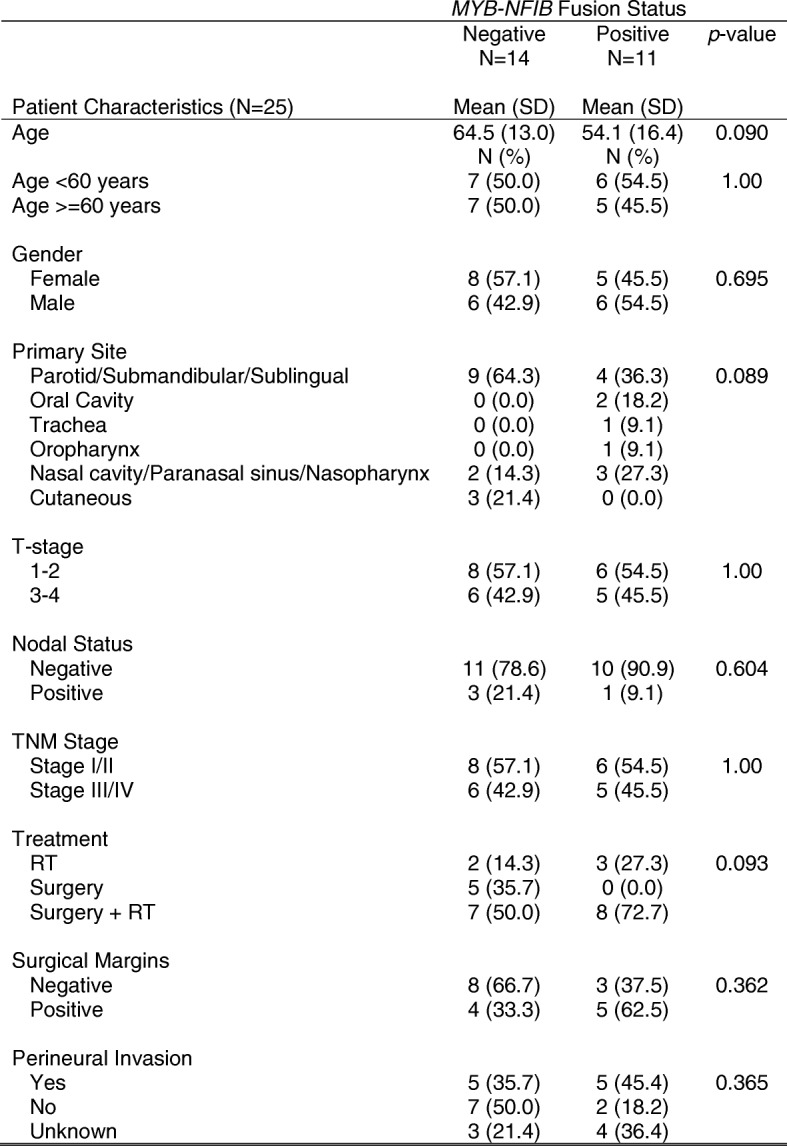
Clinocopathological characteristics of Patients Diagnosed with Adenoid Cystic Carcinoma at Tom Baker Cancer Centre 2007–2013

### RNA extraction

For each patient tissue, tumour regions were identified by pathologist review and 1 mm core punches were taken for RNA extraction. Tissue punches were de-paraffinized by xylene/ethanol treatment and total RNA was isolated using the RecoverAll™ Total Nucleic Acid Isolation kit (ThermoFisher Scientific, Waltham, MA) according to the manufacturer’s protocol with the exception that protease digestion was extended overnight at 50 °C. RNA concentration and purity was determined by Nano-Drop 2000C spectrophotometer (ThermoFisher Scientific, Waltham, MA) while RNA integrity was evaluated by Bioanalyzer 2100 using the RNA 6000 Nano kit (Agilent, Santa Clara, CA). RIN values ranged from 2.0–2.4.

### Quantitative real-time PCR analysis

RT-qPCR analysis was performed using the AB 7500 Fast PCR system (ThermoFisher Scientific, Waltham, MA). For each sample 1μg of total RNA was converted to cDNA using the SuperScript® VILO™ cDNA Synthesis Kit (ThermoFisher Scientific, Waltham, MA) following manufacturer’s protocol. To measure *MYB* 5′ and *MYB* 3′ expression, the following TaqMan gene expression primer/probes were used: Hs00920556_m1 spanning exons 3–4 and Hs00193527_m1 spanning exons 14–15 (ThermoFisher Scientific, Waltham, MA). Amplicon lengths were 76 bp and 96 bp respectively. *B2M* was used as internal reference. Each reaction consisted of 10ul of TaqMan Fast Advanced Master Mix (ThermoFisher Scientific, Waltham, MA), 2ul of cDNA template (~ 100 ng), 1ul primer/probe and 7ul nuclease-free water. All reaction assays were performed singleplex and each sample was assayed in duplicate. Pooled normal salivary gland tissue served as calibrator. *MYB* 5′ and *MYB* 3′ relative expression levels were calculated using the ΔΔ Ct method and SDS software v2.05.

### NanoString analysis

We developed a custom *MYB* and *MYB-NFIB* codeset for gene expression analysis using the NanoString nCounter Analysis System (NanoString Technologies, Seattle, WA). Fusion codesets were designed for the fully annotated *MYB-NFIB* fusion transcripts; GenBank® Accession Numbers: FJ969915.1, FJ969916.1 and FJ969917.1 with reporter probes designed to span the *MYB* and *NFIB* exon breakpoints of each fusion transcript. In addition *MYB* 5′ and *MYB* 3′ codesets were also designed to detect *MYB* 5′ and *MYB* 3′ gene expression imbalance. The complete codeset list is presented in Table 2. For each sample 300 ng of total RNA served as input. Probeset hybridization and counting were carried out according to the manufacturer’s protocol on the nCounter Prep Station and nCounter Digital Analyzer. Analysis was conducted using the NanoString nSolver Analysis Software v 2.6. Background subtraction was determined from the mean of eight ERCC negative control probes. Relative variant transcript presence in a tumour was quantified by dividing *MYB-NFIB* variant counts by *MYB* 5′ counts and presented as a percentage of total *MYB* expression. Target gene raw counts were normalized to the geometric mean counts of the following six reference genes: *RPLP0*, *POLR2A*, *HPRT1*, *GUSB*, *TBP* and *PGK1* in order to correct for RNA quality and input*.*

**Table 2:**
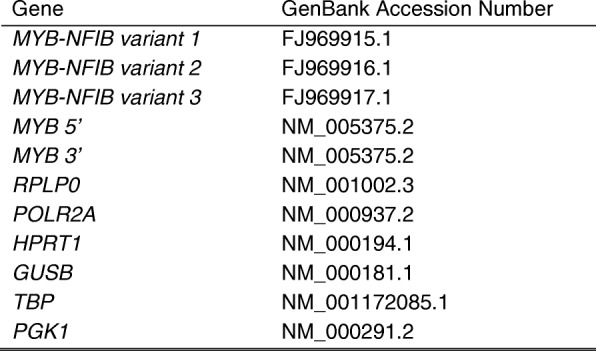
Nanostring codeset gene list

### Fusion transcript sequencing

Sanger sequencing was performed to confirm the presence of *MYB-NFIB* fusion transcripts detected by NanoString assay. cDNA derived from fusion positive samples was PCR amplified to detect *MYB-NFIB* variant 1 (MYB exon15-NFIB exon11) and *MYB-NFIB* variant 2 (MYB exon15-NFIB exon12) using the following primers: *MYB* exon15 5′-AATACCCAACTGTTCACGCA-3′, *NFIB* exon11 5′-CCTCACTGGTACTGGGGTAT-3′ and *NFIB* exon12 5′-TGGACATTGGCCGGTAAGAT-3′. Fusion transcript amplicons were confirmed on an Agilent Bioanalyzer 2100 instrument with a DNA 1000 lab chip (Agilent Technologies, Santa Clara, CA). PCR products were purified and directly sequenced on an AB 3130 Genetic Analyzer using Big Dye Terminator chemistry (ThermoFisher Scientific, Waltham, MA). Sequence traces were assembled and analyzed using Sequencing Analysis software v5.2.

### Immunohistochemistry

Tissue microarray was constructed from formalin-fixed paraffin embedded specimens. 4 micron thick sections were cut from the TMA block and de-paraffinized in xylene, rinsed in ethanol, and rehydrated. Heat-induced epitope retrieval was performed by heating slides to 121 °C in a citrate-based buffer (pH 6) Target Retrieval Solution (DAKO, Mississauga, ON, Canada) for 3 min in a de-cloaking chamber (Biocare Medical, Concord, CA, USA). Slides were stained using a Dako Autostainer Link 48 and incubations were performed at room temperature. 10 minute incubation of peroxidase block (DAKO Envision™ System) followed by 15 min protein block (Signal Stain, Cell Signaling Technology, Dancers, MA, USA) was performed to inhibit endogenous peroxidase activity and non-specific antibody binding. Slides were washed with Tris-buffered saline containing 0.05% Tween-20 (TBST, DAKO) and then incubated for 30 min with Signal Stain protein block containing 1:1000 dilution of mouse anti-MYB monoclonal antibody, clone D-7 (Santa Cruz Biotechnology, Santa Cruz, CA, USA). After three washes, goat anti-mouse horseradish peroxidase (HRP)-conjugated secondary antibody from the DAKO EnVision™ + System was applied for 30 min. Slides were again washed in TBST and treated for 5 min with TSA-Plus Cy5 tyramide signal amplification reagent (1:50, PerkinElmer, Woodbridge, ON, Canada). Incubation with rabbit anti-pan-cytokeratin antibody for 30 min followed by 1:200 dilution of Alexa-555 conjugated goat anti-rabbit antibody (Life Technologies, Burlington, ON, Canada) allows the identification of tumor cells; diamidino-2-phenylindole (DAPI) (Life Technologies) was used for nuclear identification. After three washes in TBST, the TMA slides were mounted with Pro Long™ Gold anti-fade mounting medium and stored at 4 °C overnight to set before scanning.

### Image acquisition

Automated image acquisition was performed using an Aperio Scanscope FL (Aperio Inc., Vista, CA, USA). Seamless high-resolution slide images were acquired using the Scanscope FL 8/10-bit monochrome TDI line-image capture camera using filters specific for DAPI to define the nuclear compartment, Cy3 to define cytokeratin in the tumor cytosolic compartment, and Cy5 to define the target antibody MYB. Images were then analyzed using the Indica Labs HALO program version 2.0.1145.14. Briefly, a tumor-specific mask was generated to distinguish the cancer cells from surrounding stromal tissue by thresholding the pan-cytokeratin images. All images were processed using this optimal threshold value and all subsequent image manipulations involved only image information from the masked area. TMA spots were validated and included in analysis if: 1) > 200 pan-cytokeratin positive cells per TMA spot and 2) *>* 50% of the image was usable (i.e. not compromised due to overlapping or out of focus tissue). Unusable areas within each image were manually cropped so that they were excluded from the final analysis.

### Statistical analysis

Clinical data were transferred from Excel to Stata S/E Version 13 (StataCorp. 2013. *Stata Statistical Software: Release 13*. College Station, TX: StataCorp LP) for analysis. Categorical variables were expressed as a frequency and percentage; patient age was expressed as mean and standard deviation. The student’s t-test for age and chi-square test for categorical variables (or Fisher’s exact test where appropriate) was used to test for differences in *MYB-NFIB* fusion status (negative vs positive). Overall survival (OS) was defined as the date of diagnosis to the date of death or last follow-up visit, with patients censored at their last follow-up visit. Disease-free survival (DFS) was defined as the date of diagnosis to the date of relapse, progression, death, or last follow-up visit and similarly censored at last follow-up visit. The Kaplan-Meier method was used to estimate 3-year OS and DFS. The Log-Rank test was used to test the equality of survivor functions between *MYB* fusion negative and *MYB* positive patients. A *p*-value < 0.05 was considered to be statistically significant.

## Results

### MYB 5′-3′ gene expression imbalance detected by NanoString

**Fig. 1:**
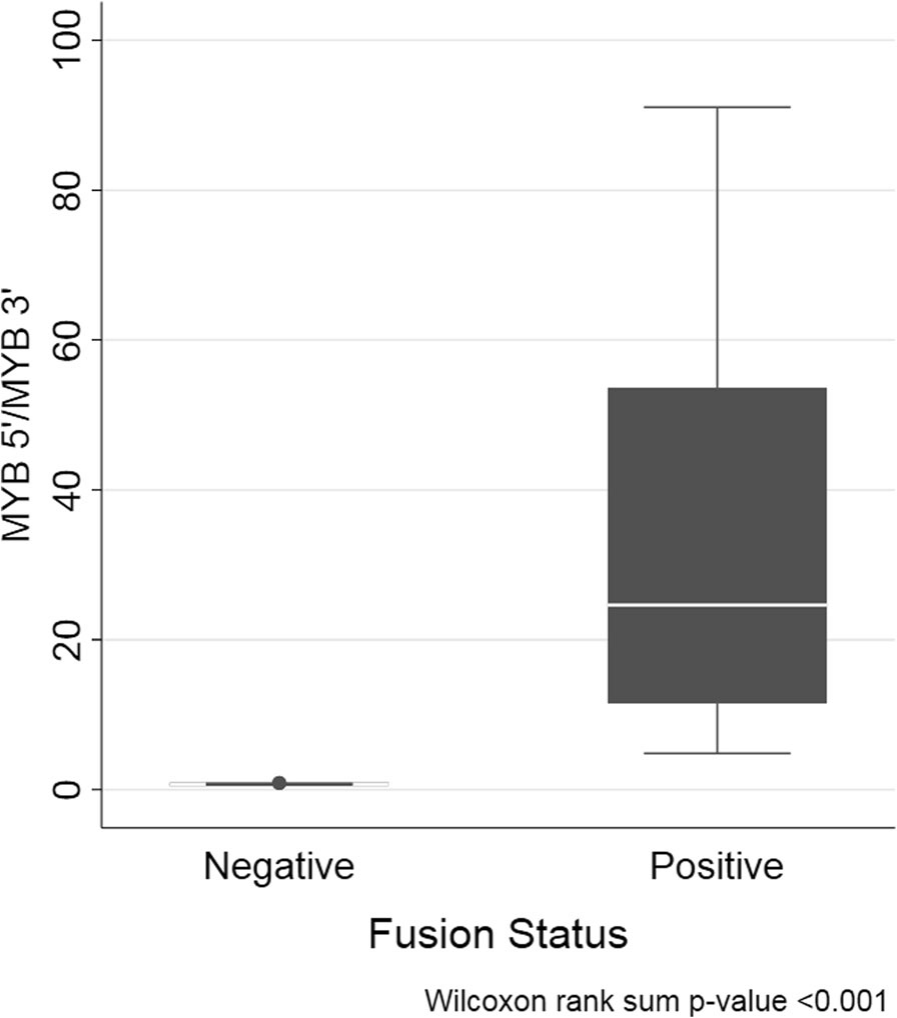
Box plots representing *MYB* 5′/3′ transcript ratios in *MYB* fusion negative ACC and *MYB* fusion positive ACC using NanoString digital probe-based technology

NanoString gene expression profiling was performed on 25 unique ACC tumours (identified as ACC1, ACC2….ACC25) and 12 normal matched tissues using a custom-designed *MYB* fusion gene panel containing *MYB* 5′ and *MYB* 3′ probes as well as probes specific for three annotated fusion variants, termed *MYB-NFIB* variants 1, 2 and 3. *MYB* transcript was over expressed in ACC versus normal tissue and we observed that 11/25 (44%) ACCs expressed *MYB* 5′-*MYB* 3′ imbalances. ACCs 1–10 and ACC 22 expressed large 5′/3′ imbalances, strongly suggestive of the expression of *MYB* fusion transcripts in these tumours (Table 3). The putative *MYB* fusion-positive ACCs had *MYB* 5′:*MYB* 3′ ratio values ranging from 4.84–91.04 (median = 24.6; interquartile range = 14.9–44.3) whereas putative fusion-negative ACCs had significantly lower ratios ranging from 0.64–0.88 (median = 0.72; interquartile range = 0.67–0.74) (Fig. 1). The normal tissues tested had generally much lower *MYB* expression counts than ACC tissues and were shown to be negative for *MYB* 5′-3′ imbalances. Normal tissue median *MYB* 5′:*MYB* 3′ ratio was 0.89 (Additional file 1: Table S1).

**Table 3:** *MYB* 5′/*MYB* 3′ and *MYB-NFIB* variant gene expression by NanoString digital counting

Sample	*MYB* 5’	*MYB* 3’	*MYB* 5′/*MYB* 3’	*MYB-NFIB* variant 1	*MYB-NFIB* variant 2	*MYB-NFIB* variant 3	Fusion Status
ACC 1	11779.23	335.18	35.14	3522.52 (0.299)	1354.35 (0.114)	1	pos
ACC 2	7278.12	1503.18	4.84	2678.01 (0.367)	1498.96 (0.161)	1	pos
ACC 3	8657.07	967.26	8.95	3746.86 (0.432)	2731.55 (0.315)	1.02	pos
ACC 4	4530.06	180.88	25.04	2.57	4527.15 (0.999)	1	pos
ACC 5	8220.75	401.62	20.47	1	1	1	pos
ACC 6	4716.43	86.66	54.42	1	3.69	4.67	pos
ACC 7	16054.49	1390.11	11.55	1	4.39	1	pos
ACC 8	15812.18	173.68	91.04	1.18	9.88	1	pos
ACC 9	7394.09	402.95	18.35	1.01	1.09	1.01	pos
ACC 10	5285.36	214.78	24.61	1	3.18	4.91	pos
ACC 11	571.27	763.47	0.75	3.33	5.06	24.11	neg
ACC 12	17289.7	19617.38	0.88	1	14.15	1	neg
ACC 13	8255.09	12033.66	0.69	3.54	6.14	1	neg
ACC 14	13302.14	16149.15	0.82	3.04	3.98	1	neg
ACC 15	9259.06	13802.03	0.67	1	6.53	1	neg
ACC 16	242.92	332.99	0.73	1	4.61	1	neg
ACC 17	166.49	258.98	0.64	3.89	1.49	1.49	neg
ACC 18	12660.4	18399.28	0.69	1.22	7.45	1.22	neg
ACC 19	394.09	526.29	0.75	1.51	2.32	1	neg
ACC 20	8974.13	13486	0.67	1	5.49	1.34	neg
ACC 21	11148.45	15087.98	0.74	6.68	3.53	4.32	neg
ACC 22	15449.27	288.09	53.63	1	79.75 (0.005)	1	pos
ACC 23	3631.33	5070.83	0.72	1.48	3.78	1	neg
ACC 24	3668.04	5465.07	0.67	1.15	1	1	neg
ACC 25	2286.75	2975.37	0.77	1.06	1.06	1.06	neg

**Fig. 2:**
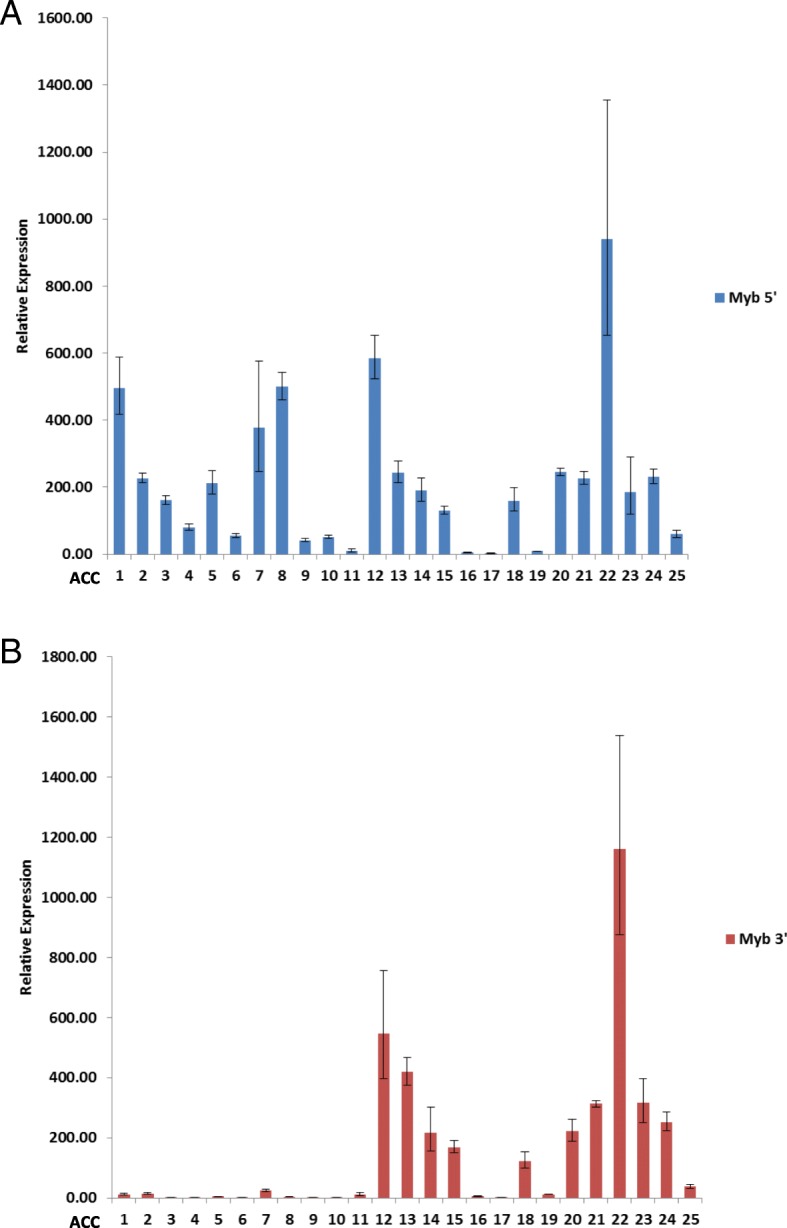
Expression of *MYB* 5′ and *MYB* 3′ transcripts in 25 ACC cases by RT-qPCR. Relative expression of *MYB* 5′ transcripts (**a**) using primer/probe pairs that amplify coding exons 3–4 and *MYB* 3′ transcripts (**b**) using primer/probe pairs that amplify coding exons 14–15. Results are represented as fold increase expression relative to pooled normal tissue

To confirm the results of the NanoString *MYB* 5′-3′ assay we performed a similar *MYB* 5′-3′ gene expression analyses using quantitative real-time PCR. Two sets of TaqMan primer/probes were used targeting exon junctions 3–4 and 14–15. RT-qPCR analysis was performed on ACCs 1–25 and results showed that *MYB* 5′ gene expression was much higher than *MYB* 3′ expression in ACCs 1–10 (Fig. 2). All remaining tumour samples including ACC 22 were shown to have comparable *MYB* 5′ and 3′ relative gene expression levels. With the exception of ACC 22, these results mirrored our findings with the NanoString assay.

**Fig. 3:**
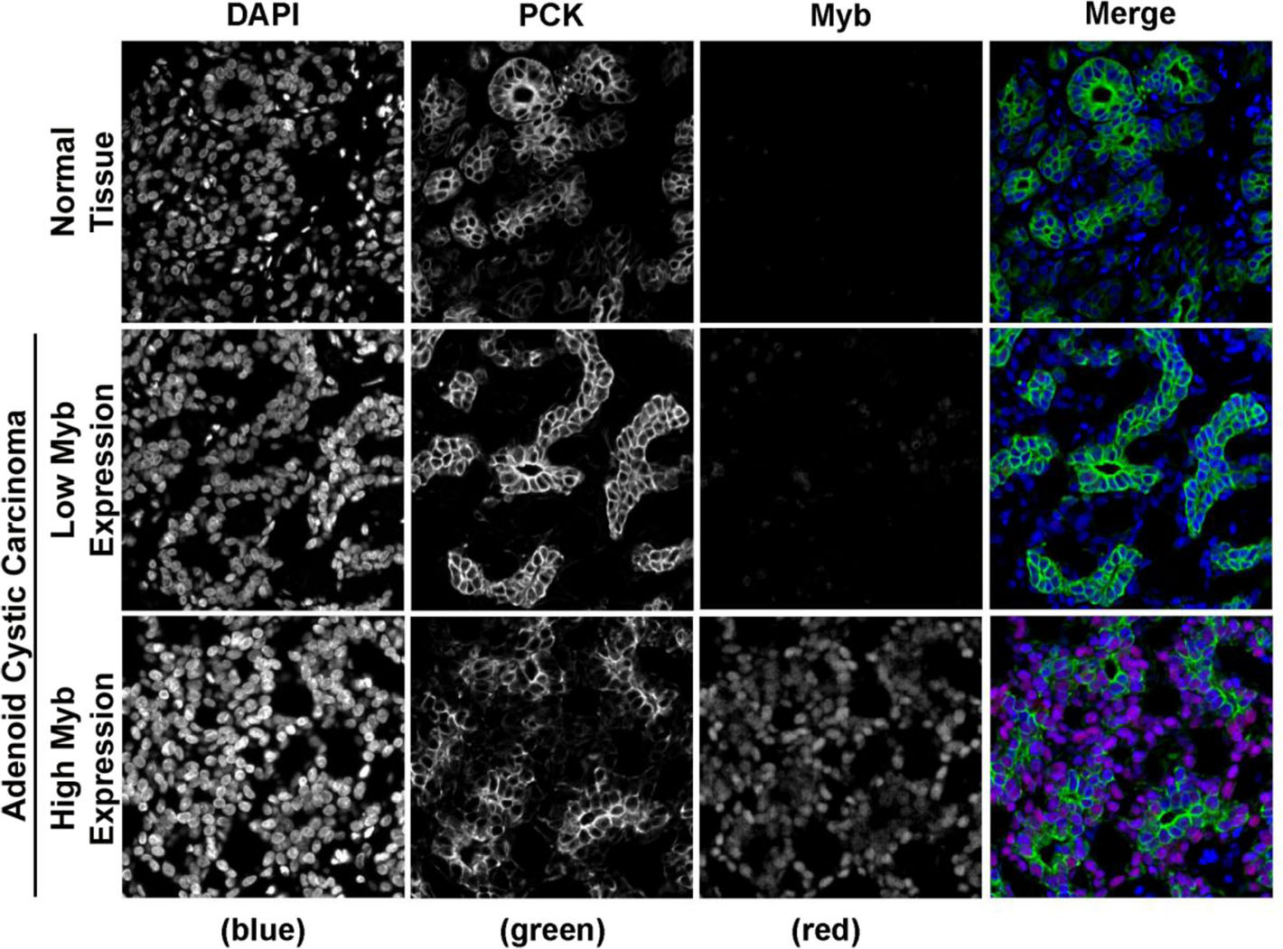
MYB protein expression. Representative fluorescence microscopy micrographs of paraffin-embedded normal tissue and example of ACC tissues expressing different amount of MYB protein. TMA were subjected to anti-MYB (red) and anti-Pan cytokeratin (green) antibodies indirect immunofluorescence and nuclear counterstaining with DAPI (blue)

MYB protein expression was assessed by immunohistochemistry on tissue microarrays constructed from the 25 ACC and 12 normal tissues in our study cohort. The analysis revealed strong MYB nuclear staining in ACCs expressing high *MYB* transcript. Like-wise, tumours that had lower *MYB* mRNA expression had corresponding lower MYB protein levels. Normal tissues had near undetectable MYB protein expression (Fig. 3).

### Specific MYB-NFIB variant transcript detection by NanoString

In addition to *MYB* 5′ and 3′ transcript imbalances, the NanoString assay detected expression of *MYB-NFIB* variants 1 and 2 (MYB exon15-NFIB exon11 and MYB exon15-NFIB exon12) but did not detect expression of *MYB-NFIB* variant 3 in any of our study samples (Table 3). These targeted *MYB-NFIB* variants were only present in ACC tumours that exhibited strong *MYB* 5′/*MYB* 3′ gene expression imbalances. Five of the eleven (45%) putative fusion-positive ACCs (including ACC 22) expressed one or more of these variants, confirming their fusion status. In ACCs that expressed both variants 1 and 2 (ACCs 1–3), fusion variant 1 (MYB exon15-NFIB exon11) was the predominant chimeric transcript present accounting for 30–43% of total *MYB* transcript (Additional file 2: Figure S1). Noticeably, in these cases, *MYB* 3′ counts did not account for the difference in total *MYB* 5′ counts and summed *MYB-NFIB* variant 1 and variant 2 counts. This may suggest that other *MYB* fusion transcripts possibly involving *NFIB* or other 3′ gene partners are present in these tumours. Interestingly, ACC 4 expressed only *MYB-NFIB* variant 2 transcript (MYB exon 15-NFIB exon 12) and at nearly 100% of total *MYB* transcript expression. However ACC 4 also had *MYB* 3′ counts of 180.88, suggesting low-level expression of wild-type *MYB* transcript. Conversely, ACC 22 expressed variant 2 but at very low levels, less than 1% of *MYB* transcript. Given the high degree of *MYB* 5′-*MYB* 3′ imbalance observed in this sample, these results strongly suggest the co-expression of additional *MYB* fusion transcripts.

### Sequencing analysis

**Fig. 4:**
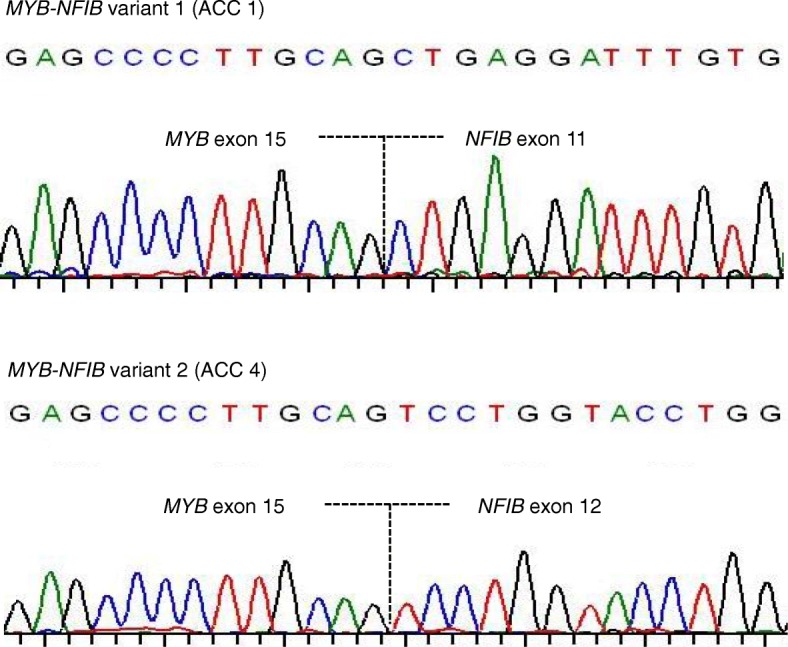
Representative sequencing profiles of *MYB-NFIB* variants 1 and 2 detected in fusion positive ACC patients. *MYB* nucleotide sequence and exon data is based on transcript accession number ENST00000341911 in the Ensembl database and *NFIB* sequence and exon information is based on Ensembl transcript accession number ENST00000397581

Using cDNA, direct sequencing was performed on ACCs 1–4 and ACC 22 to verify the identity of *MYB-NFIB* variants as detected by the NanoString fusion panel. Sequence analysis confirmed that the NanoString fusion probe assay was able to correctly identify the species of fusion variant reported in each tumour (Fig. 4). In the case of ACC 22, sequencing not only confirmed *MYB-NFIB* variant 2 but also showed the additional robust presence of a *MYB* exon 16-*NFIB* exon 12 fusion transcript confirming the expression of at least one additional *MYB* fusion variant in this tumour (Additional file 3: Figure S2). This variant has previously been reported by Mitani et al [7].

### MYB fusion and patient survival

**Fig. 5:**
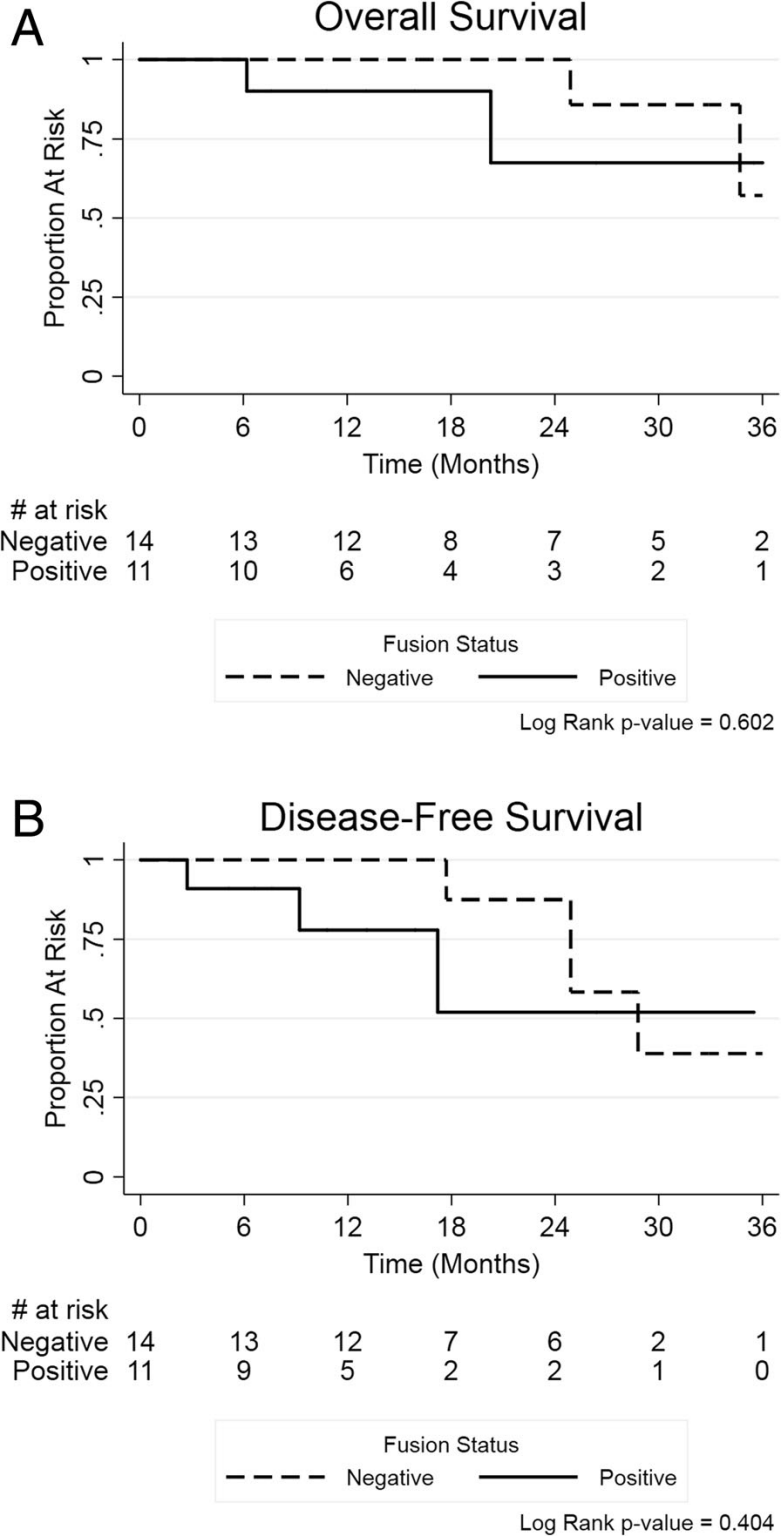
Kaplan-Meier survival outcomes by *MYB* fusion status

To examine if there were any clinical outcome associations with fusion status we performed Kaplan-Meier analyses. No significant differences were observed in overall survival between fusion positive (3 year OS: 67.5% (95% CI = 16.2, 91.9%) and fusion negative patients (3 year OS: 57.1% (95% CI = 7.6, 88.6%). Neither was there any association in disease-free survival between the two groups (fusion positive 3 year DFS: 52% (95% CI = 8.3, 84.2%; fusion negative 3 year DFS: 38.9% (95% CI = 6.3, 72.4%) (Fig. 5). Similarly, when examining clinicopathological characteristics, we found no statistically significant differences between patients according to *MYB* fusion status (Table 1).

## Discussion

In the present pilot study, we investigated the feasibility of employing NanoString technology to detect *MYB-NFIB* fusion transcripts in archival FFPE adenoid cystic carcinoma tissues. We designed a single-tube multiplex custom NanoString panel targeting direct fusion breakpoints of three known *MYB-NFIB* variants as well as a probeset for MYB 5′/3′ imbalance interrogation. The benefit of this combinatorial approach is that the *MYB* 5′/3′ testing strategy does not require previous knowledge of fusion partners or fusion breakpoints. Similar strategies have been used to detect relevant fusion transcripts in various other cancers [15, 16]. Using this methodology we were able to detect *MYB* 5′/3′ imbalanced mRNA expression levels in nearly half (44%) the cases in our study cohort, similar to what other studies have found in fresh frozen ACC tissue using quantitative real time PCR [7, 14]. These cases expressed high *MYB* 5′ counts but had considerably lower *MYB* 3′ counts, a hallmark strongly suggestive of the presence of *MYB* fusion transcripts. Indeed, five of these ACC samples were positive for either *MYB-NFIB* variant 1 and/or variant 2 by direct fusion probe detection and cDNA sequencing. The remaining six cases were negative for the specific variants in our assay but positive for *MYB* transcript imbalance indicating the likelihood of additional *MYB-NFIB* transcripts or perhaps the presence of *MYB* transcripts with other 3′ fusion partners or even overexpression of truncated *MYB* splice variants [6, 7, 17–19].

We in turn tested and compared our *MYB* 5′/3′ NanoString assay results with RT-qPCR and found agreement in all but one sample, ACC 22, as noted. This result was inconsistent with the high *MYB* 5′:*MYB* 3′ ratio we observed in ACC 22 by NanoString. We postulate that this discrepancy is most likely due to the design of the NanoString *MYB* 3′ probe which is located within the 3’UTR of the *MYB* gene whereas the RT-qPCR 3′ TaqMan primer/probe spans exons 14–15 of the *MYB* gene. Thus, a *MYB* fusion junction 3′ distal to exon 15 would not be detected by this RT-qPCR design. Our results support this postulation as observed by the very low expression of *MYB-NFIB* variant 2, an exon 15 fusion (*MYB* exon 15-*NFIB* exon 12) coupled with the sequence data identifying co-expression of a *MYB* exon 16-*NFIB* exon 12 transcript. This highlights the importance of assay design and the consideration for future *MYB* 5′/3′ assays to position the 3′ primer/probe within the *MYB* 3’UTR for comprehensive fusion breakpoint screening.

A significant benefit of NanoString technology is the capability to produce highly sensitive and specific target molecule counts. In the context of ACC genomics, this level of gene expression quantification can allow for molecular sub-classification with respect to *MYB* fusion gene expression. ACC, like many other head and neck cancers is highly heterogeneous, and as our results demonstrate fusion transcripts are often co-expressed [20–22]. When multiple fusion transcripts are present in a tumour, determination of abundant and rare fusion transcript expression may reveal biological associations with differential phenotypic behaviors or responses to therapy. This may be pertinent in ACC where *MYB* translocations are the most common molecular feature. The NanoString assay provides a multiplexed quantitative assessment of *MYB* fusion expression that exceeds the qualitative nature of conventional methodologies such as PCR and FISH, thus permitting such levels of investigation. Indeed, a recent global gene expression study found that ACCs with *MYB* or *MYBL1* fusions occurring after exon 11 (termed “long fusions”) clustered together and distinctly apart from tumours with fusions at exons 8 or 9 [10]. This data suggests differential transcriptional profiles in ACC associated with specific fusion breakpoints. It is worth noting that this study was performed using fresh ACC tissues and fusion variants annotated and confirmed by conventional RT-PCR and Sanger sequencing.

We have previously published the patient characteristics and survival outcomes of the entire cohort of 60 consecutive patients with ACC [23]. Due to the retrospective nature of this study, we could only locate 25 patients with analyzable tissue samples in this 7-year period. The cohort with *MYB* fusion includes less female patients, has younger median age and more positive margin status, however, these differences may be due to the small sample size. More patients received surgery alone in the *MYB* fusion negative cohort, although treatments received did not differ statistically. Most patients in both cohorts presented with node negative and earlier T- stage disease, representative of a typical ACC patient population. Overall, none of the known prognostic factors showed statistically significant difference between the two cohorts [23]. Our study did not show differences in survival outcomes between MYB fusion positive and negative cohorts. This finding is similar to previously published studies that found numerically better but no statistically significant differences in survival with *MYB* positive ACC [24, 25]. Larger studies are required to confirm the prognostic value of *MYB* further.

In addition to increasing sample size, assay methodology improvements should be considered for future studies. Our multiplexed assay was designed to test a single 5′ and 3′ probe locus for *MYB* imbalance expression. Although able to detect the presence of fusions, the assay as designed would fail to report if either probe reaction were to fail. Having a minimum of three 5′ and three 3′ *MYB* probe sets would mitigate this potential issue. Furthermore, averaging values obtained from these probe reactions would provide more precise transcript imbalance measurement as well as improving the accuracy of fusion variant expression relative to *MYB* 5′ expression. For a comprehensive analysis, the assay in its present form would need to expand to include probes for all *MYB-NFIB* transcript variants reported to date, requiring extensive literature review and experimentation in order to accurately annotate and validate fusion junctions. At the time of conducting this pilot study, sequence information for only three variants was publicly available on the NCBI database. Another limitation of our study was the inability to evaluate the performance of the NanoString assay in fresh tissue as compared to archived FFPE tissue. Fresh frozen tissue was unavailable to our study however previous other gene expression studies have shown good correlation [13, 26, 27].

## Conclusion

In summary our pilot study demonstrates the feasibility of employing multiplexed digital profiling for *MYB-NFIB* variant transcripts in archival tissue using NanoString technology. This detection methodology is ideal for multiplex fusion analysis using FFPE tissue and can overcome the limitations imposed by past studies that required fresh tissue for similar investigations. Despite the small size of our study, we believe the NanoString assay described provides researchers a promising tool for the accurate detection and quantification of *MYB* fusions in patients with adenoid cystic carcinoma.

## Additional files

Additional file 1: *MYB* 5':*MYB* 3' gene expression ratios in matched normal ACC tissue. (DOCX 12 kb)

Additional file 2: *MYB-NFIB* variant 1 and 2 transcript expression relative to overall *MYB* expression. (PPTX 527 kb)

Additional file 3: *MYB* exon 16-*NFIB* exon 12 fusion in ACC 22 detected by sequencing. (PPTX 176 kb)

## Data Availability

The datasets used and/or analyzed during the current study are available from the corresponding author on reasonable request.

## Abbreviations

ACC: Adenoid cystic carcinoma
cDNA: Complementary DNA
DAPI: Diamidino-2-phenylindole
DFS: Disease-free survival
FFPE: Formalin-fixed paraffin embedded
FISH: Fluorescence in situ hybridization
OS: Overall survival
RIN: RNA integrity number
RT-PCR: Reverse transcription-polymerase chain reaction
