# Supplementary material for: MYB-NFIB gene fusions identified in archival adenoid cystic carcinoma tissue employing NanoString analysis: an exploratory study
Source: Diagn Pathol. 2019 Jul 13;14:78. doi: 10.1186/s13000-019-0855-8 (PMC6626627; doi:10.1186/s13000-019-0855-8)
Supplement: Supplementary file 1 — MYB 5':MYB 3' gene expression ratios in matched normal ACC tissue. (DOCX 12 kb) [file 13000_2019_855_MOESM1_ESM.docx]

Supplementary Table 1. *MYB* 5’:*MYB* 3’ gene expression ratios in matched normal ACC tissue.

| Sample | *MYB* 5' | *MYB* 3' | *MYB* 5':*MYB* 3' |
| --- | --- | --- | --- |
| ACC 1N | 75.53 | 102.49 | 0.74 |
| ACC 2N | 443.84 | 485.33 | 0.91 |
| ACC 3N | 35.33 | 33.89 | 1.04 |
| ACC 5N | 25.4 | 16.98 | 1.50 |
| ACC 11N | 103.6 | 135.31 | 0.77 |
| ACC 12N | 180.74 | 206.75 | 0.87 |
| ACC 13N | 23.29 | 25.01 | 0.93 |
| ACC 15N | 364.92 | 483.97 | 0.75 |
| ACC 16N | 24.6 | 27.41 | 0.90 |
| ACC 20N | 24.06 | 28.23 | 0.85 |
| ACC 22N | 53.55 | 86.74 | 0.62 |
| ACC 24N | 19.03 | 10.83 | 1.76 |
