# Supplementary figures and images for: MYB-NFIB gene fusions identified in archival adenoid cystic carcinoma tissue employing NanoString analysis: an exploratory study
Source: Diagn Pathol. 2019 Jul 13;14:78. doi: 10.1186/s13000-019-0855-8 (PMC6626627; doi:10.1186/s13000-019-0855-8)

## Slide 1
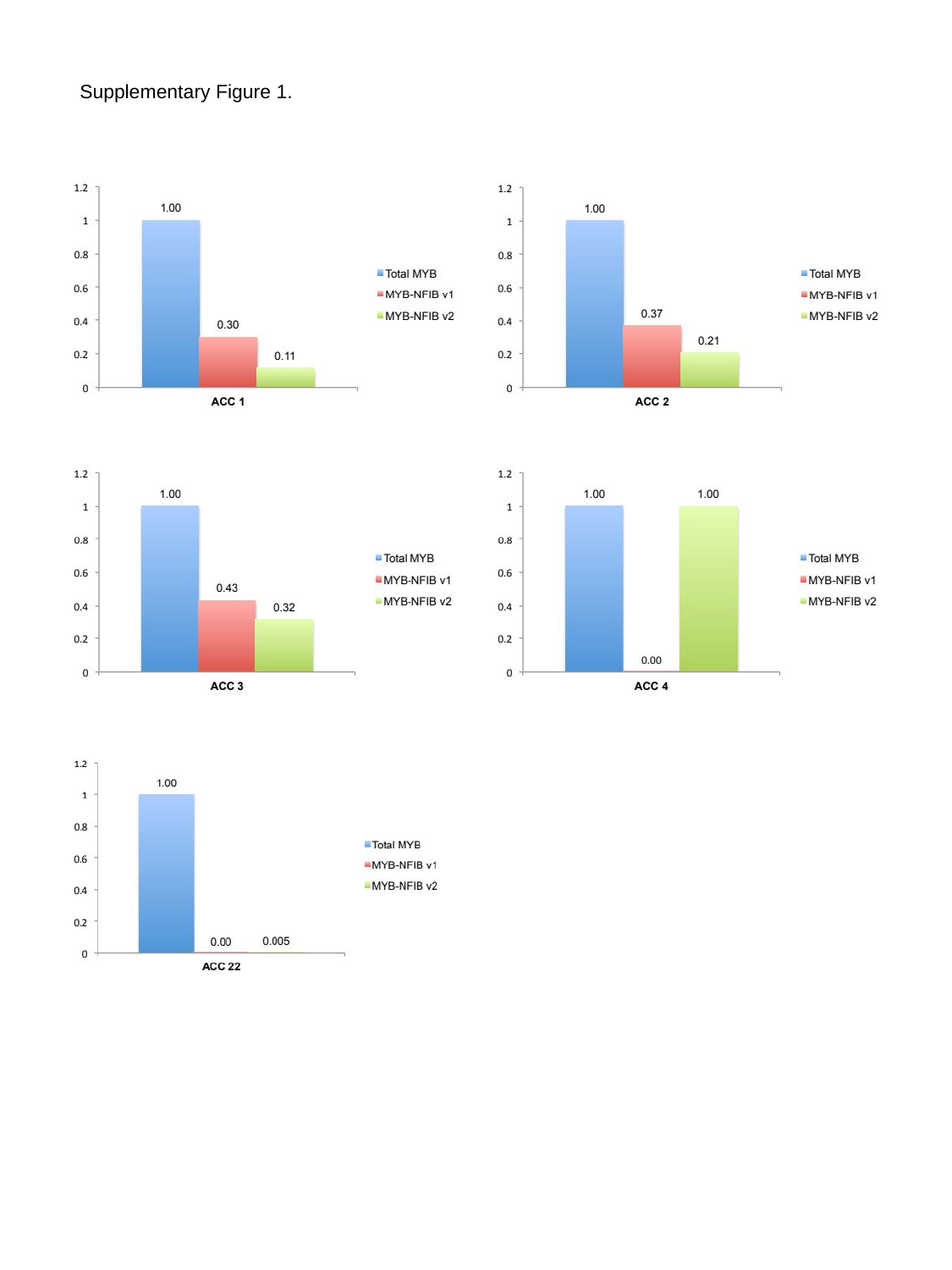

Supplementary Figure 1.

Supplement: Supplementary file 2 — MYB-NFIB variant 1 and 2 transcript expression relative to overall MYB expression. (PPTX 527 kb) [file 13000_2019_855_MOESM2_ESM.pptx]

## Slide 1
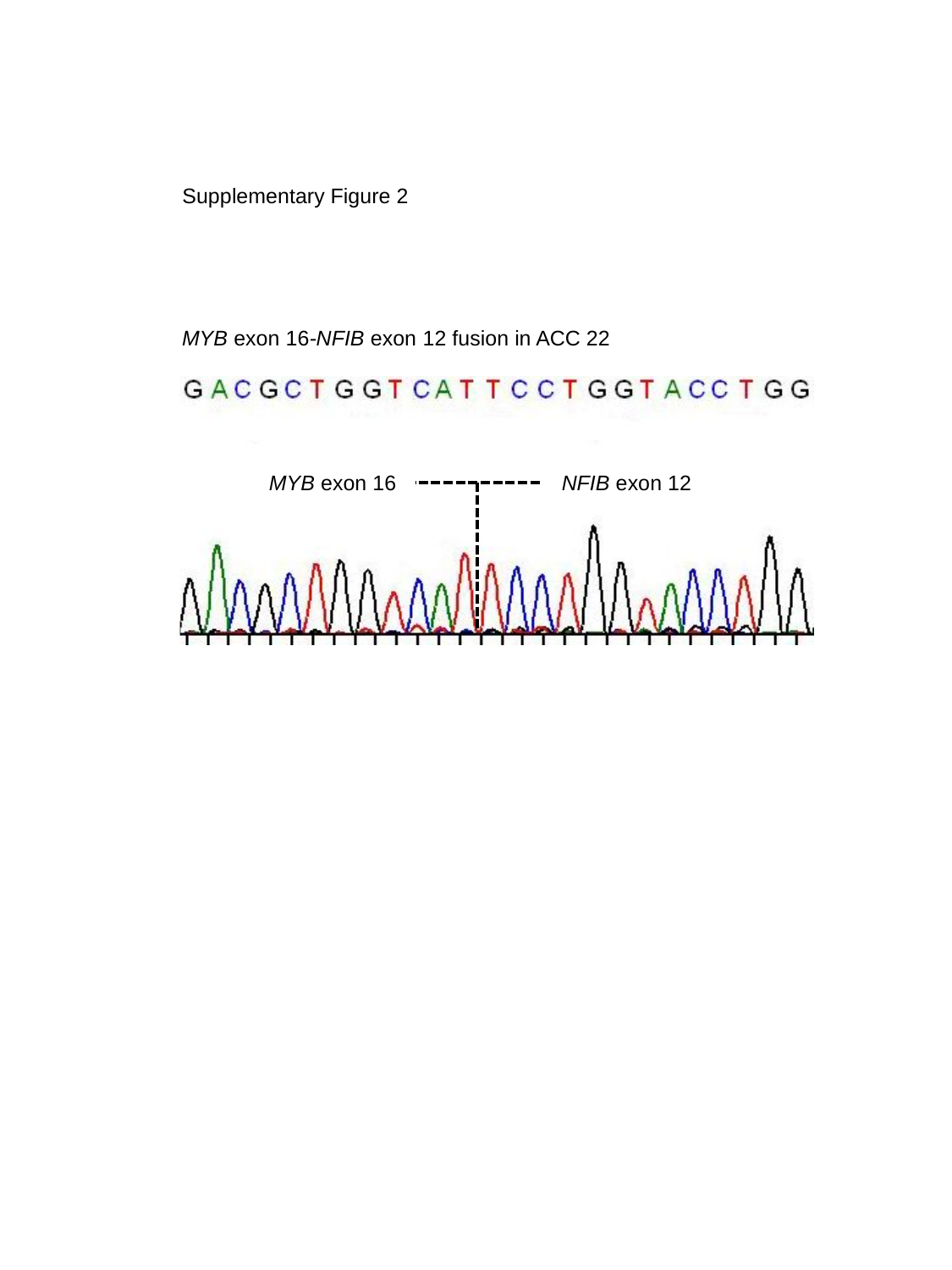

Supplementary Figure 2
MYB exon 16-NFIB exon 12 fusion in ACC 22
NFIB exon 12
MYB exon 16

Supplement: Supplementary file 3 — MYB exon 16-NFIB exon 12 fusion in ACC 22 detected by sequencing. (PPTX 176 kb) [file 13000_2019_855_MOESM3_ESM.pptx]
